# A comprehensive comparison of random forests and support vector machines for microarray-based cancer classification

**DOI:** 10.1186/1471-2105-9-319

**Published:** 2008-07-22

**Authors:** Alexander Statnikov, Lily Wang, Constantin F Aliferis

**Affiliations:** 1 Department of Biomedical Informatics, Vanderbilt University, Nashville, TN, USA; 2 Department of Biostatistics, Vanderbilt University, Nashville, TN, USA; 3 Department of Cancer Biology, Vanderbilt University, Nashville, TN, USA; 4 Department of Computer Science, Vanderbilt University, Nashville, TN, USA

## Abstract

**Background:**

Cancer diagnosis and clinical outcome prediction are among the most important emerging applications of gene expression microarray technology with several molecular signatures on their way toward clinical deployment. Use of the most accurate classification algorithms available for microarray gene expression data is a critical ingredient in order to develop the best possible molecular signatures for patient care. As suggested by a large body of literature to date, support vector machines can be considered "best of class" algorithms for classification of such data. Recent work, however, suggests that random forest classifiers may outperform support vector machines in this domain.

**Results:**

In the present paper we identify methodological biases of prior work comparing random forests and support vector machines and conduct a new rigorous evaluation of the two algorithms that corrects these limitations. Our experiments use 22 diagnostic and prognostic datasets and show that support vector machines outperform random forests, often by a large margin. Our data also underlines the importance of sound research design in benchmarking and comparison of bioinformatics algorithms.

**Conclusion:**

We found that both on average and in the majority of microarray datasets, random forests are outperformed by support vector machines both in the settings when no gene selection is performed and when several popular gene selection methods are used.

## Background

Gene expression microarrays are becoming increasingly promising for clinical decision support in the form of diagnosis and prediction of clinical outcomes of cancer and other complex diseases. In order to maximize benefits of this technology, researchers are continuously seeking to develop and apply the most accurate classification algorithms for the creation of gene expression patient profiles. Prior research suggests that among well-established and popular techniques for multicategory classification of microarray gene expression data, support vector machines (SVMs) have a predominant role, significantly outperforming k-nearest neighbours, backpropagation neural networks, probabilistic neural networks, weighted voting methods, and decision trees [1].

In the last few years substantial interest has developed within the bioinformatics community in the random forest algorithm [2] for classification of microarray and other high-dimensional molecular data [3-5]. The random forest algorithm possesses a number of appealing properties making it well-suited for classification of microarray data: (i) it is applicable when there are more predictors than observations, (ii) it performs embedded gene selection and it is relatively insensitive to the large number of irrelevant genes, (iii) it incorporates interactions between predictors, (iv) it is based on the theory of ensemble learning that allows the algorithm to learn accurately both simple and complex classification functions, (v) it is applicable for both binary and multicategory classification tasks, and (vi) according to its inventors it does not require much fine-tuning of parameters and the default parameterization often leads to excellent performance [2]. Recent work [5] reported an empirical evaluation of random forests in the cancer microarray gene expression domain and concluded that random forest classifiers have predictive performance comparable to that of the best performing alternatives (including SVMs) for classification of microarray gene expression data. In fact, the data in Table 2 of [5] suggests that random forests on average across 10 datasets slightly outperform SVMs as well as other methods. If true, this finding could be of great significance to the field, because combined with prior results about SVM performance (e.g., [1]), this suggests that random forests offer classification accuracy advantages over "best of class" classifier algorithms for this type of data.

However, closer inspection of this prior comparison [5] reveals several important data analytic biases that may have affected its conclusions: First, while the random forests were applied to datasets prior to gene selection, SVMs were applied with a subset of only 200 genes (the number 200 was chosen arbitrarily). Given that the number of optimal genes varies from dataset to dataset, and that SVMs are known to be fairly insensitive to a very large number of irrelevant genes, such application of SVMs likely biases down their performance. Second, a one-versus-one SVM algorithm was applied for the multicategory classification tasks, while it is has been shown that in microarray gene expression domain this method is inferior to other multicategory SVM methods, such as one-versus-rest [1,6]. Third, the evaluation of [5] was limited only to linear SVMs without optimizing any algorithm parameters such as the penalty parameter *C *that balances data fit with insensitivity to outliers. Fourth, the performance metric used in [5], proportion of correct classifications, is sensitive to unbalanced distribution of classes and has lower power to discriminate among classification algorithms compared to existing alternatives such as area under the ROC curve and relative classifier information [7-10]. Fifth, no statistical comparison among classifiers has been performed. Finally, the prior comparison uses a .632+ bootstrap error estimator [11] which is not the most appropriate error estimator for microarray data where powerful classifiers such as SVMs and RFs typically achieve 0 training error and the .632+ bootstrap becomes equivalent to repeated hold-out estimation that may suffer from the training-set-size bias as discussed in [12]. Furthermore, .632+ bootstrap is currently not developed for performance metrics other than proportion of correct classifications.

We hypothesize that these apparent methodological biases of prior work have compromised its conclusions and the question of whether random forests indeed outperform SVMs for classification of microarray gene expression data is not convincingly answered. In the present work we undertake a more methodologically rigorous comparison of the two algorithms to determine the relative errors when applied to a wide variety of datasets. We examine the algorithms both in the settings when no gene selection is performed and when several popular gene selection methods are used. To make our evaluation more relevant to practitioners, we focus not only on diagnostic datasets that are in general known to have strong predictive signals, but also include several outcome prediction datasets where the signals are weaker and larger gene sets are often required for optimal prediction.

## Results

### Using full set of genes

**Figure 1:**
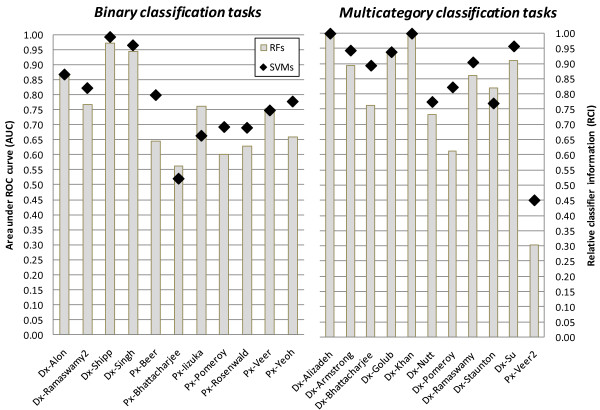
**Classification performance of SVMs and RFs without gene selection**. The performance is estimated using area under ROC curve (AUC) for binary classification tasks and relative classifier information (RCI) for multicategory tasks.

The performance results of classification prior to gene selection are shown in Figure 1 and Table 1. In total, SVMs nominally (that is, not necessarily statistically significantly) outperform RFs in 15 datasets, RFs nominally outperform SVMs in 4 datasets, and in 3 datasets algorithms perform the same. The application of permutation-based statistical comparison test with significance level α = 0.05 reveals that SVMs significantly outperform RFs in 7 datasets, while RFs do not significantly outperform SVMs in any dataset. The permutation test applied to all 22 datasets shows that SVMs statistically significantly outperform RFs on average over all datasets at the 0.05 α level (p-value of the test = 0.008). It is also worthwhile to compare both methods in terms of the average performance across datasets. The average performance of SVMs is 0.775 AUC and 0.860 RCI in binary and multicategory classification tasks, respectively. The average performance of RFs in the same tasks is 0.742 AUC and 0.803 RCI.

**Table 1:** Comparison of classification performance of SVMs and RFs without gene selection.

***Task & dataset***	***Classification performance metric***	***Classification performance***		***Nominally superior method***	***P-value***
				
		***SVM***	***RF***		
*Dx-Alon*	AUC	0.867	0.867	-	1
*Dx-Ramaswamy2*	AUC	0.821	0.767	SVM	0.409
*Dx-Shipp*	AUC	0.992	0.973	SVM	0.500
*Dx-Singh*	AUC	0.964	0.944	SVM	0.377
*Px-Beer*	AUC	0.798	0.646	SVM	**0.032**
*Px-Bhattacharjee*	AUC	0.519	0.561	RF	0.546
*Px-Iizuka*	AUC	0.663	0.763	RF	0.061
*Px-Pomeroy*	AUC	0.692	0.600	SVM	0.235
*Px-Rosenwald*	AUC	0.689	0.629	SVM	0.140
*Px-Veer*	AUC	0.747	0.754	RF	0.867
*Px-Yeoh*	AUC	0.777	0.660	SVM	**0.006**

*Dx-Alizadeh*	RCI	1.000	1.000	-	1
*Dx-Armstrong*	RCI	0.944	0.894	SVM	0.658
*Dx-Bhattacharjee*	RCI	0.895	0.763	SVM	**0.015**
*Dx-Golub*	RCI	0.939	0.934	SVM	1
*Dx-Khan*	RCI	1.000	1.000	-	1
*Dx-Nutt*	RCI	0.775	0.733	SVM	0.498
*Dx-Pomeroy*	RCI	0.823	0.611	SVM	**0.031**
*Dx-Ramaswamy*	RCI	0.905	0.861	SVM	**0.010**
*Dx-Staunton*	RCI	0.770	0.819	RF	0.249
*Dx-Su*	RCI	0.958	0.910	SVM	**0.004**
*Px-Veer2*	RCI	0.451	0.304	SVM	**0.004**

The performance is estimated using area under ROC curve (AUC) for binary classification tasks and relative classifier information (RCI) for multicategory tasks. See subsection "Statistical comparison among classifiers" for the description of statistical test employed to compute reported p-values. P-values shown with boldface denote statistically significant differences between classification methods at the 0.05 α level.

### Using gene selection

**Figure 2:**
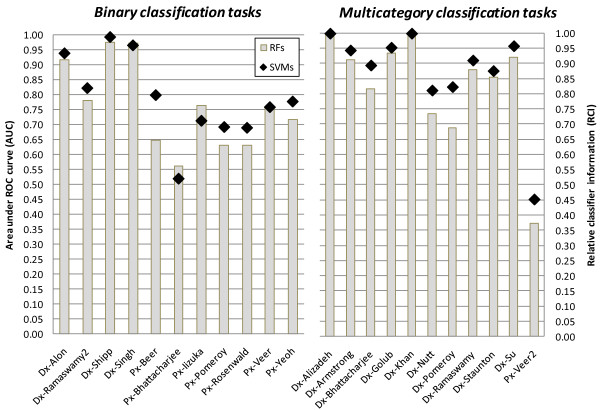
**Classification performance of SVMs and RFs with gene selection**. The performance is estimated using area under ROC curve (AUC) for binary classification tasks and relative classifier information (RCI) for multicategory tasks.

Six classification performance estimates have been produced for each classifier and dataset (5 estimates corresponding to various gene selection methods and one estimate corresponding to using no gene selection). In Figure 2 and Table 2 we present a comparison based on the best performing gene selection method for each algorithm and dataset combination under the operating assumption that practitioners will optimize choice of the gene selection method for each dataset separately (using cross-validation or other suitable protocols). The results in Figure 2 and Table 2 thus better mirror the actual practice of data analysis.

**Table 2:** Comparison of classification performance of SVMs and RFs with gene selection.

***Task & dataset***	***Classification performance metric***	***Classification performance***		***Nominally superior method***	***P-value***
				
		***SVM***	***RF***		
*Dx-Alon*	AUC	0.938	0.917	SVM	0.626
*Dx-Ramaswamy2*	AUC	0.821	0.781	SVM	0.624
*Dx-Shipp*	AUC	0.992	0.975	SVM	0.502
*Dx-Singh*	AUC	0.964	0.972	RF	0.812
*Px-Beer*	AUC	0.798	0.648	SVM	**0.016**
*Px-Bhattacharjee*	AUC	0.519	0.561	RF	0.550
*Px-Iizuka*	AUC	0.713	0.763	RF	0.750
*Px-Pomeroy*	AUC	0.692	0.629	SVM	0.506
*Px-Rosenwald*	AUC	0.689	0.631	SVM	0.128
*Px-Veer*	AUC	0.758	0.754	SVM	0.954
*Px-Yeoh*	AUC	0.777	0.716	SVM	0.082

*Dx-Alizadeh*	RCI	1.000	1.000	-	1
*Dx-Armstrong*	RCI	0.944	0.911	SVM	0.624
*Dx-Bhattacharjee*	RCI	0.895	0.817	SVM	0.125
*Dx-Golub*	RCI	0.953	0.934	SVM	1
*Dx-Khan*	RCI	1.000	1.000	-	1
*Dx-Nutt*	RCI	0.812	0.733	SVM	0.220
*Dx-Pomeroy*	RCI	0.823	0.688	SVM	0.079
*Dx-Ramaswamy*	RCI	0.911	0.880	SVM	0.066
*Dx-Staunton*	RCI	0.876	0.856	SVM	0.626
*Dx-Su*	RCI	0.958	0.922	SVM	0.078
*Px-Veer2*	RCI	0.451	0.371	SVM	0.262

The performance is estimated using area under ROC curve (AUC) for binary classification tasks and relative classifier information (RCI) for multicategory tasks. See subsection "Statistical comparison among classifiers" for the description of statistical test employed to compute reported p-values. P-values shown with boldface denote statistically significant differences between classification methods at the 0.05 α level.

According to the results in Figure 2 and Table 2, in 17 datasets SVMs nominally outperform RFs, in 3 datasets RFs nominally outperform SVMs, and in 2 datasets algorithms perform the same. Furthermore, SVMs outperform RFs statistically significantly (at the 0.05 α level) in 1 dataset. There is no dataset where RFs outperform SVMs with statistically significant difference. The permutation test applied to all 22 datasets shows that SVMs statistically significantly outperform RFs on average over all datasets at the 0.05 α level (p-value of the test = 0.001). A comparison of the average performance across datasets also suggests superiority of SVMs: the average performance of SVMs is 0.787 AUC and 0.875 RCI in binary and multicategory classification tasks, respectively; while the average performance of RFs in the same tasks is 0.759 AUC and 0.828 RCI.

The number of genes selected on average across 10 cross-validation training sets is provided in Table 3. We note that in the present comparison we focus exclusively on classification performance and do not incorporate number of selected genes in the comparison metrics because there is no well-defined trade-off between number of selected genes and classification performance in the datasets studied. Nevertheless, the detailed classification results for all gene selection methods, classifiers, and datasets are provided in the Additional File 1.

**Table 3:** Number of genes selected for each microarray dataset and gene selection method.

***Task & dataset***	***No gene selection***	***RFE***	***RFVS1***	***RFVS2***	***KW***	***S2N***
*Dx-Alizadeh*	4026	12	62	73	19	15
*Dx-Alon*	2000	105	16	3	15	13
*Dx-Armstrong*	11225	74	709	57	106	48
*Dx-Bhattacharjee*	12600	289	27	15	1864	653
*Dx-Golub*	5327	12	456	336	42	4
*Dx-Khan*	2308	28	17	18	15	11
*Dx-Nutt*	10367	1598	126	101	476	926
*Dx-Pomeroy*	5920	186	34	16	70	435
*Dx-Ramaswamy*	15009	3346	966	411	8248	10277
*Dx-Ramaswamy2*	13247	1576	12	4	4129	1364
*Dx-Shipp*	5469	8	15	6	13	89
*Dx-Singh*	10509	157	58	21	22	38
*Dx-Staunton*	5726	169	152	73	93	97
*Dx-Su*	12533	2429	845	320	1318	1927
*Px-Beer*	7129	201	15	7	953	1380
*Px-Bhattacharjee*	12600	21	46	7	138	61
*Px-Iizuka*	7070	103	38	7	168	185
*Px-Pomeroy*	7129	70	29	13	445	439
*Px-Rosenwald*	7399	2338	124	27	3201	3897
*Px-Veer*	24188	1056	124	20	5388	4405
*Px-Veer2*	24188	491	149	39	1194	1764
*Px-Yeoh*	12240	1187	21	6	3077	1869

Average number of genes selected over 10 cross-validation training sets.

## Discussion

The results presented in this paper illustrate that SVMs offer classification performance advantages compared to RFs in diagnostic and prognostic classification tasks based on microarray gene expression data. We emphasize that when it comes to clinical applications of such models, because the size of the patient populations is typically very large, even very modest differences in performance (e.g., at the order of 0.01 AUC/RCI or even less) can result in very substantial differences in total clinical outcomes (e.g., number of life-years saved) [13].

The reasons for superior classification performance of one universal approximator classifier over the other in a domain where the generative functions are unknown are not trivial to decipher [2,14]. We provide here as a starting point two plausible explanations supported by theory and a simulation experiment (in Additional File 2). We note that prior research has established that linear decision functions capture very well the underlying distributions in microarray classification tasks [15,16]. In the following two paragraphs we first demonstrate that for such functions SVMs may be less sensitive to the choice of input parameters than RFs and then explain why SVMs model linear decision functions more naturally than RFs.

The simulation experiment described in Additional File 2 demonstrates high degree of sensitivity of RFs to the values of input parameters *mtry *(i.e., number of genes randomly selected at each node) and *ntree *(i.e., number of trees) even in the case of linear decision function when complicated decision surface modelling is not required. The experiment shows that the choice of RF parameters creates large variation in the classifier performance whereas the choice of the main SVM parameter has only minor effects on the error. In practical analysis of microarrays this means that finding the RFs with optimal error for the dataset may involve extensive model selection which in turn opens up the possibility for overfitting given the small sample sizes in validation datasets.

A second plausible explanation is that decision trees used as base learners in the RF algorithm cannot learn exactly many linear decision functions in the finite case. Specifically, if the generative linear decision function is not orthogonal to the coordinate axes, then a decision tree of infinite size is required to represent this function without error [17]. The voted decision function in RFs approximates linear functions based on rectangular partitioning of the input space, and this "staircase" approximation can capture a linear function exactly when the number of decision trees can grow without bound (assuming that each tree is of finite size). SVMs on the other hand use linear classifiers and thus can model such functions naturally, using a small number of free parameters (i.e., bounded by the available sample size).

We note that regardless of the specific reasons why RFs may have larger error on average in this domain, it is still important to be aware of the empirical performance differences when considering which classifier to use for building molecular signatures. It may take several years before the precise reasons of differences in empirical error are thoroughly understood, and in the meantime the empirical advantages and disadvantages of methods should be noted first by practitioners.

Data analysts should also be aware of a limitation of RFs imposed by its embedded random gene selection. In order for a RF classification model to overcome the trap of large variance, one has to use a large number of trees and build trees based on a large number of genes. The exact values of these parameters depend on both the complexity of the classification function and the number of genes in a microarray dataset. Therefore, in general, it is advisable to optimize these parameters by nested cross-validation that accounts for the variability of the random forest model (e.g., the selected parameter configuration is the one that performs best on average over multiple validation sample sets).

Finally, it is worthwhile to mention the work by Segal [18] who questioned Breiman's empirical demonstration of the claim that random forests do not overfit as the number of trees grows [2]. In short, Segal showed that there exist some data distributions where maximal unpruned trees used in the random forests do not achieve as good performance as the trees with smaller number of splits and/or smaller node size. Thus, application of random forests in general requires careful tuning of the relevant classifier parameters. These observations may suggest future improvements of RF-related analysis protocols.

## Conclusion

The primary contribution of the present work is that we conducted the most comprehensive comparative benchmarking of random forests and support vector machines to date, using 22 diagnostic and outcome prediction datasets. Our hypothesis that in previously reported work, research design limitations may have biased the comparison of classifiers in favour of random forests, was verified. After removing these benchmarking limitations, we found that, both on average and in the majority of microarray datasets, random forests exhibit larger classification error than support vector machines both in the settings when no gene selection is performed and when several gene selection methods are used.

The quest for high performance classifiers with microarray gene expression and other "omics" data is ongoing. Random forests have appealing theoretical and practical characteristics, however our experiments show that currently they do not exhibit "best of class" performance. Our data also points to methodological limitations of prior evaluations and thus emphasizes the importance of careful design of bioinformatics algorithm evaluation studies.

## Methods

### Microarray datasets and classification tasks

Gene expression microarray datasets used in the present work are described in Table 4. All 22 datasets span the domain of cancer; 14 datasets correspond to diagnostic tasks (and denoted with prefix "Dx") and 8 are concerned with clinical outcome prediction (and denoted with "Px"). Out of 22 datasets, 11 are binary classification tasks, while the other 11 are multicategory tasks with 3–26 classes. The datasets contain 50–308 samples and 2,000–24,188 variables (genes) after data preparatory steps described in [1]. All diagnostic datasets were obtained from [1] and from the links given in the primary study for each dataset. Similarly, all prognostic datasets were obtained from the links given in the primary study for each dataset. A list of references to the primary study for each dataset is provided in the Additional File 3. Notice that the dataset collection used in this work contains all datasets from the prior comparison [5].

**Table 4:** Gene expression microarray datasets used in this study.

***Task & dataset***	***Number of classes***	***Number of genes***	***Number of samples***	***Prediction task***
*Dx-Alizadeh*	3	4026	62	Diffuse large B-cell lymphoma, follicular lymphoma, chronic lymphocytic leukemia
*Dx-Alon*	2	2000	62	Colon tumors and normal tissues
*Dx-Armstrong*	3	11225	72	AML, ALL and mixed-lineage leukemia (MLL)
*Dx-Bhattacharjee*	5	12600	203	4 lung cancer types and normal tissues
*Dx-Golub*	3	5327	72	Acute myelogenous leukemia (AML), acute lymphoblastic leukemia (ALL) B-cell and ALL T-cell
*Dx-Khan*	4	2308	83	Small, round blue cell tumors of childhood
*Dx-Nutt*	4	10367	50	4 malignant glioma types
*Dx-Pomeroy*	5	5920	90	5 human brain tumor types
*Dx-Ramaswamy*	26	15009	308	14 various human tumor types and 12 normal tissue types
*Dx-Ramaswamy2*	2	13247	76	Metastatic and primary tumors
*Dx-Shipp*	2	5469	77	Diffuse large B-cell lymphomas and follicular lymphomas
*Dx-Singh*	2	10509	102	Prostate tumor and normal tissues
*Dx-Staunton*	9	5726	60	9 various human tumor types
*Dx-Su*	11	12533	174	11 various human tumor types
*Px-Beer*	2	7129	86	Lung adenocarcinoma survival
*Px-Bhattacharjee*	2	12600	62	Lung adenocarcinoma 4-year survival
*Px-Iizuka*	2	7070	60	Hepatocellular carcinoma 1-year recurrence-free survival
*Px-Pomeroy*	2	7129	60	Medulloblastoma survival
*Px-Rosenwald*	2	7399	240	Non-Hodgkin lymphoma survival
*Px-Veer*	2	24188	97	Breast cancer 5-year metastasis-free survival
*Px-Veer2*	3	24188	115	Breast cancer 5-year metastasis-free survival, metastasis within 5 years, germline BRCA1 mutation
*Px-Yeoh*	2	12240	233	Acute lymphocytic leukemia relapse-free survival

The reference paper for each dataset is provided in the Additional File 3.

### Cross-validation design

We used 10-fold cross-validation to estimate the performance of the classification algorithms. In order to optimize algorithm parameters, we used another "nested" loop of cross-validation by further splitting each of the 10 original training sets into smaller training sets and validation sets. For each combination of the classifier parameters, we obtained cross-validation performance and selected the best performing parameters inside this inner loop of cross-validation. Next, we built a classification model with the best parameters on the original training set and applied this model to the original testing set. Details about the "nested cross-validation" procedure can be found in [19,20]. Notice that the final performance estimate obtained by this procedure will be unbiased because each original testing set is used only once to estimate performance of a single classification model that was built by using training data exclusively.

### Support vector machine classifiers

Several theoretical reasons explain the superior empirical performance of SVMs in microarray data: e.g., they are robust to the high variable-to-sample ratio and large number of variables, they can learn efficiently complex classification functions, and they employ powerful regularization principles to avoid overfitting [1,21,22]. Extensive applications literature in text categorization, image recognition and other fields also shows the excellent empirical performance of this classifier in many more domains. The underlying idea of SVM classifiers is to calculate a maximal margin hyperplane separating two classes of the data. To learn non-linearly separable functions, the data are implicitly mapped to a higher dimensional space by means of a kernel function, where a separating hyperplane is found. New samples are classified according to the side of the hyperplane they belong to [22]. Many extensions of the basic SVM algorithm can handle multicategory data. The "one-versus-rest" SVM works better for multi-class microarray data [1,6], so we adopted this method for the analysis of multicategory datasets in the present study. In summary, this approach involves building a separate SVM model to classify each class against the rest, and then predicting the class of a new sample using the SVM model with the strongest vote.

We used SVM implementation in the *libSVM *software library [23] with polynomial kernel. Recall that the SVM polynomial kernel can be defined as: *K*(*x*, *y*) = (γ·*x*^T^*y *+ *r*)^*d*^, where *x *and *y *are samples with gene expression values and γ, *r*, *d *are kernel parameters. The parameters γ and *r *were set to default value 1. The kernel degree *d *together with the SVM penalty parameter *C *were optimized by nested cross-validation over *d *values {1, 2, 3} and *C *values {0.01, 1, 100}.

### Random forest classifiers

Random forests (RF) is a classification algorithm that uses an ensemble of unpruned decision trees, each of which is built on a bootstrap sample of the training data using a randomly selected subset of variables [2]. As mentioned in the Background section, this algorithm possesses a number of properties making it an attractive technique for classification of microarray gene expression data.

We employed the state-of-the-art implementation of RF available in the R package *randomForest *[24]. This implementation is based on the original Fortran code authored by Leo Breiman, the inventor of RFs. Following the suggestions of [24,25] and , we considered different parameter configurations for the values of *ntree *= {500, 1000, 2000} (number of trees to build), *mtryFactor *={0.5, 1, 2} (a multiplicative factor of the default value of *mtry *parameter denoting the number of genes randomly selected at each node; by default *mtry *= $\sqrt{number\cdot of\cdot genes}$), and *nodesize *= 1 (minimal size of the terminal nodes of the trees in a random forest) and selected the best-performing configuration by nested cross-validation. Note that the above parameter values are also consistent with the recommendations of the study [5].

### Gene selection methods

Even though both SVM and RF classifiers are fairly insensitive to very large number of irrelevant genes, we applied the following widely used gene selection methods in order to further improve classification performance:

• Random forest-based backward elimination procedure RFVS [5]: The RFVS procedure involves iteratively fitting RFs (on the training data), and at each iteration building a random forest after discarding genes with the smallest importance values. The returned subset of genes is the one with the smallest out-of-bag error. We used the *varSelRF *implementation of the RFVS method developed by its inventors and applied it with the recommended parameters: *ntree *= 2000, *mtryFactor *= 1, *nodesize *= 1, *fraction.dropped *= 0.2 (a parameter denoting fraction of genes with small importance values to be discarded during backward elimination procedure), and *c.sd *= 0 (a factor that multiplies the standard deviation of error for stopping iterations and choosing the best performing subset of genes). We refer to this method as "RFVS1."

• RFVS procedure as described above, except for *c.sd *= 1 (denoted as "RFVS2"): This method differs from RFVS1 in that it performs statistical comparison to return the smallest subset of genes with performance statistically indistinguishable from the nominally best one.

• SVM-based recursive feature elimination method RFE [26]: This is a state-of-the-art procedure for gene selection from microarray data that involves iteratively fitting SVM classification models (on the training data) by discarding the genes with the small impact on classification and selecting the smallest subset of genes that participate in the best performing classification model (as assessed in the validation data). Even though RFE was originally introduced as a method for binary classification problems, it can be trivially extended to multiclass case by using binary SVM models in "one-versus-rest" fashion (e.g., see [27]). Finally, to be comparable with the RFVS method, we used the fraction of genes that are discarded in the iterative SVM models equal to 0.2.

• Backward elimination procedure based on univariate ranking of genes with "signal-to-noise" ratio [1,21,28] (denoted as "S2N"): This procedure first ranks all genes according their signal-to-noise value with the response variable, and then performs backward elimination using SVM classifier (fit on the training set and evaluated on the validation set) to determine the best performing smallest subset of genes. Similarly to RFE and RFVS, we perform backward elimination by discarding 0.2 proportion of genes at each iteration.

• Backward elimination procedure based on univariate ranking of genes with Kruskal-Wallis one-way non-parametric ANOVA [1] (denoted as "KW"): This procedure is applied similarly to the S2N method except for it uses different univariate ranking of genes.

We emphasize that all gene selection methods were applied during cross-validation utilizing only the training data and splitting it into a smaller training and validation set if necessary.

### Classification performance evaluation metrics

We used two classification performance metrics. For binary tasks, we used the area under the ROC curve (AUC) which was computed from continuous outputs of the classifiers (distances from separating hyperplane for SVMs and outcome probabilities for RFs) [8]. For multicategory tasks, where classical AUC is inapplicable, we employed the relative classifier information (RCI) [7]. RCI is an entropy-based measure that quantifies how much the uncertainty of a decision problem is reduced by a classifier relative to classifying using only the prior probabilities of each class. We note that both AUC and RCI are more discriminative than the accuracy metric (also known as proportion of correct classifications) and are not sensitive to unbalanced distributions [7-10]. Both AUC and RCI take values on [0, 1], where 0 denotes worst possible classification and 1 denotes perfect classification.

### Statistical comparison among classifiers

When comparing two classifiers, it is important to assess whether the observed difference in classification performance is statistically significant or simply due to chance. We assessed significance of differences in classification performance in individual datasets or in all datasets on average using a non-parametric permutation test [29] based on the theory of [30]. The null hypothesis of this test is no difference between performance of SVM and RF classifiers. The test was applied with 100,000 permutations and two-sided p-values were computed as described in [29]. We used a significance level α = 0.05 for this test.

## Authors' contributions

Conceived and designed the experiments: AS, LW, CFA. Performed the experiments: AS. Analyzed the results of experiments: AS, LW, CFA. Wrote the paper: AS, CFA. All authors read and approved the final manuscript.

## Supplementary Material

Additional file 1 Results for all gene selection methods, classifiers, and datasets. Click here for file

Additional file 2 Simulation experiment demonstrating sensitivity of random forests to input parameters. Click here for file

Additional file 3 Complete information about microarray datasets used in the study. Click here for file
