# Supplementary material for: A comprehensive comparison of random forests and support vector machines for microarray-based cancer classification
Source: BMC Bioinformatics. 2008 Jul 22;9:319. doi: 10.1186/1471-2105-9-319 (PMC2492881; doi:10.1186/1471-2105-9-319)
Supplement: Additional file 1 — Results for all gene selection methods, classifiers, and datasets. [file 1471-2105-9-319-S1.pdf]

Additional File 1:
Results for all gene selection methods, classifiers and datasets.

Dx-**Su**

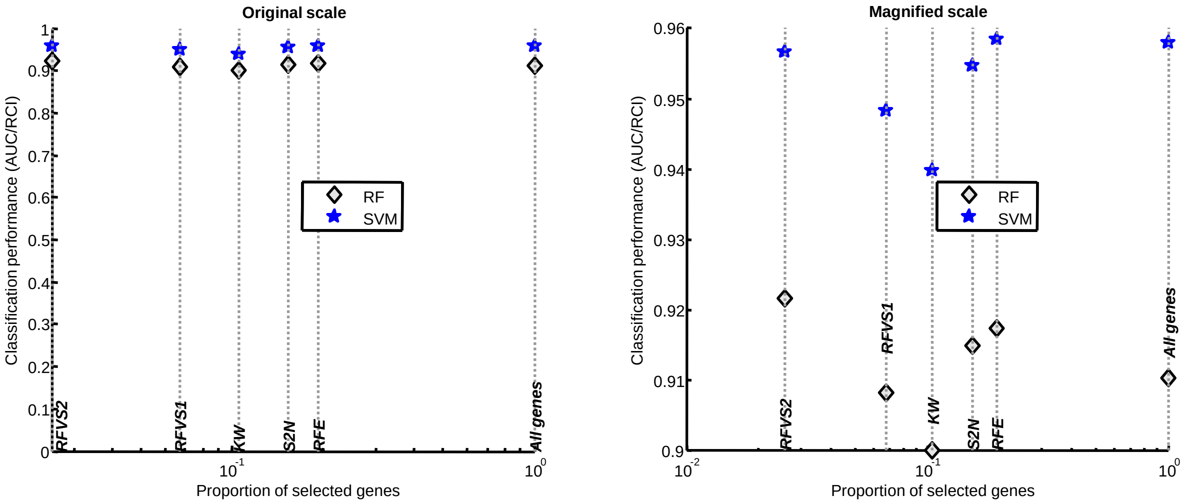

Dx-**Ramaswamy**

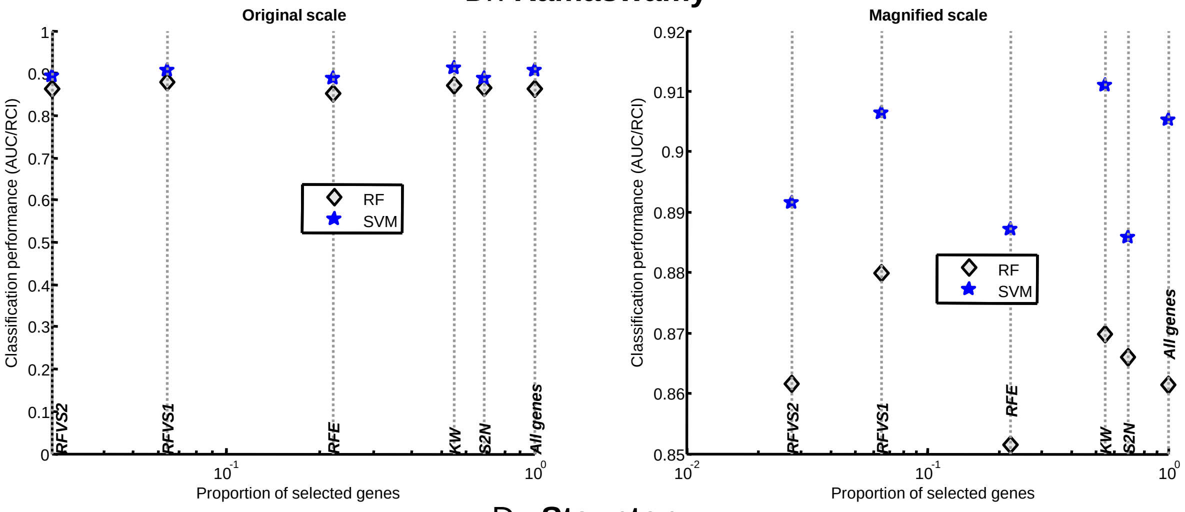

Dx-**Staunton**

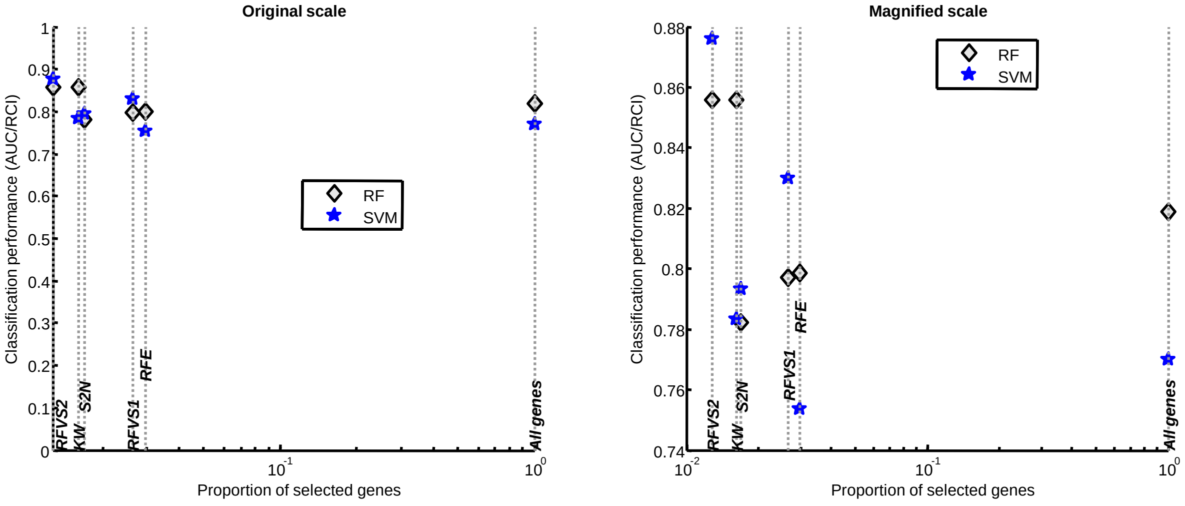

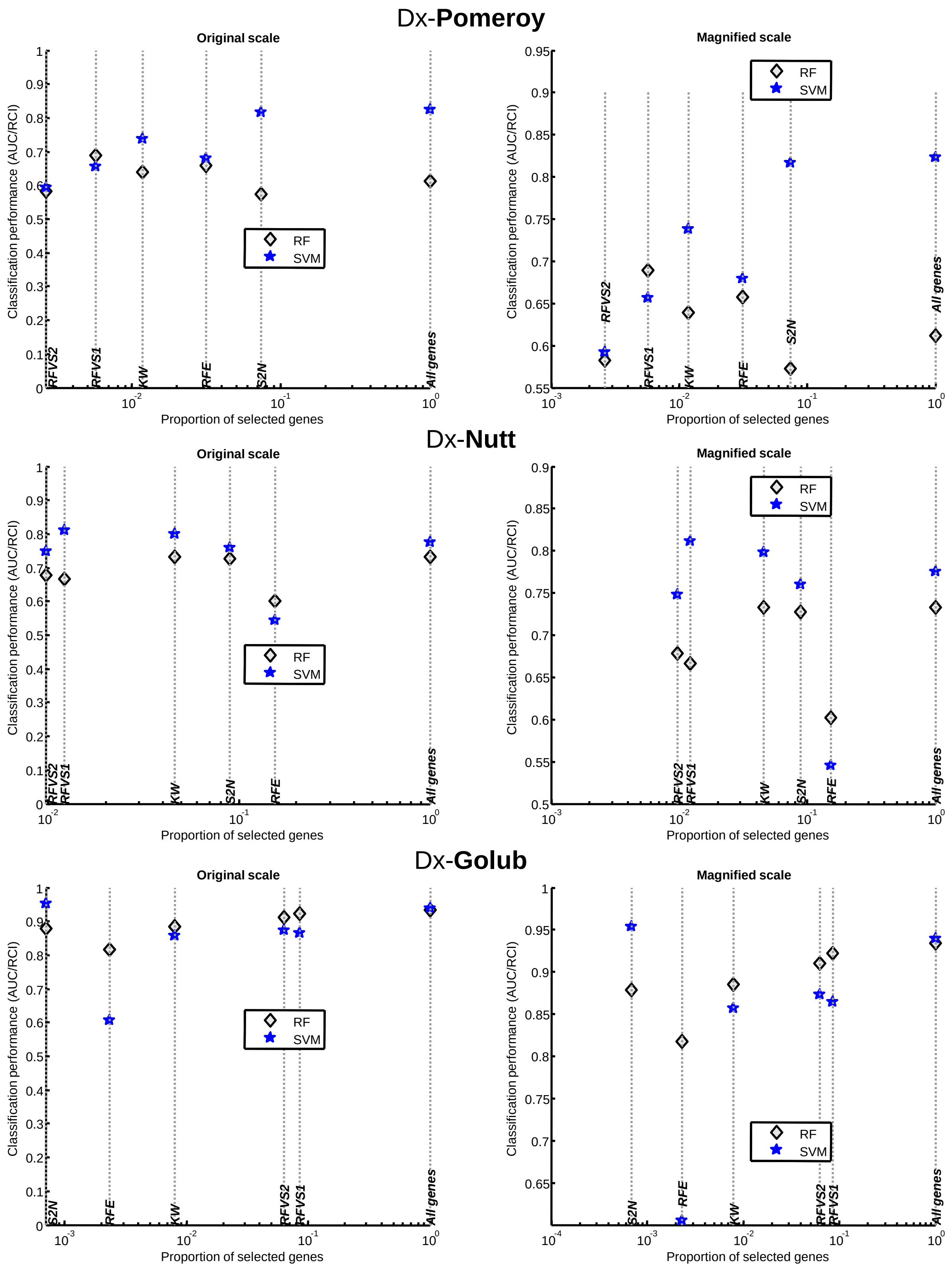
Dx-Pomeroy
Original scale
Magnified scale
Classification performance (AUC/RCI)
Proportion of selected genes
RF
SVM
RFVS2
RFVS1
KW
RFE
S2N
All genes
Dx-Nutt
Original scale
Magnified scale
Classification performance (AUC/RCI)
Proportion of selected genes
RF
SVM
RFVS2
RFVS1
KW
S2N
RFE
All genes
Dx-Golub
Original scale
Magnified scale
Classification performance (AUC/RCI)
Proportion of selected genes
RF
SVM
S2N
RFE
KW
RFVS2
RFVS1
All genes

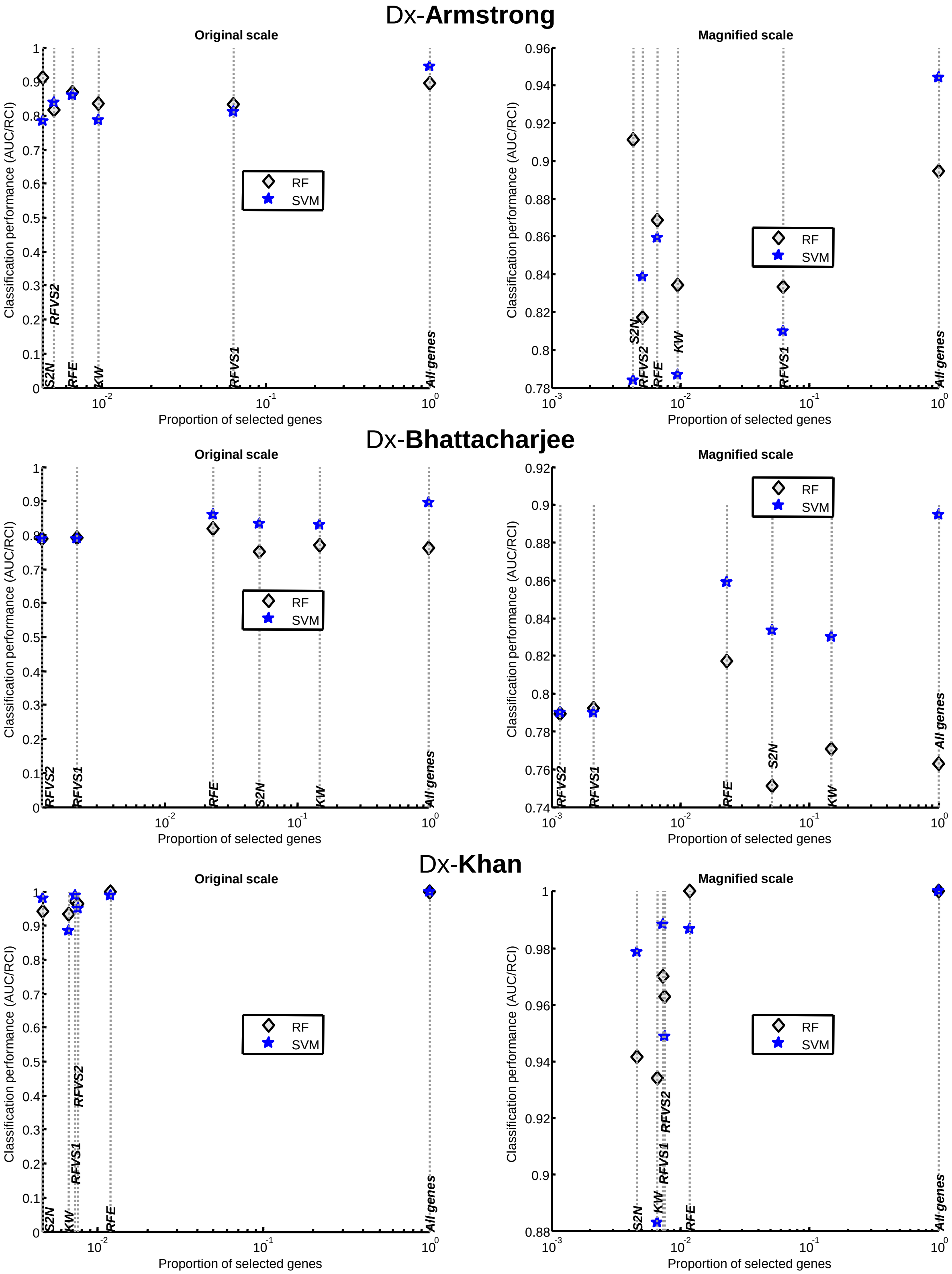
Dx-Armstrong
Original scale
Magnified scale
Dx-Bhattacharjee
Original scale
Magnified scale
Dx-Khan
Original scale
Magnified scale
Classification performance (AUC/RCI)
Proportion of selected genes
RF
SVM
S2N
RFVS1
RFVS2
RFE
KW
All genes

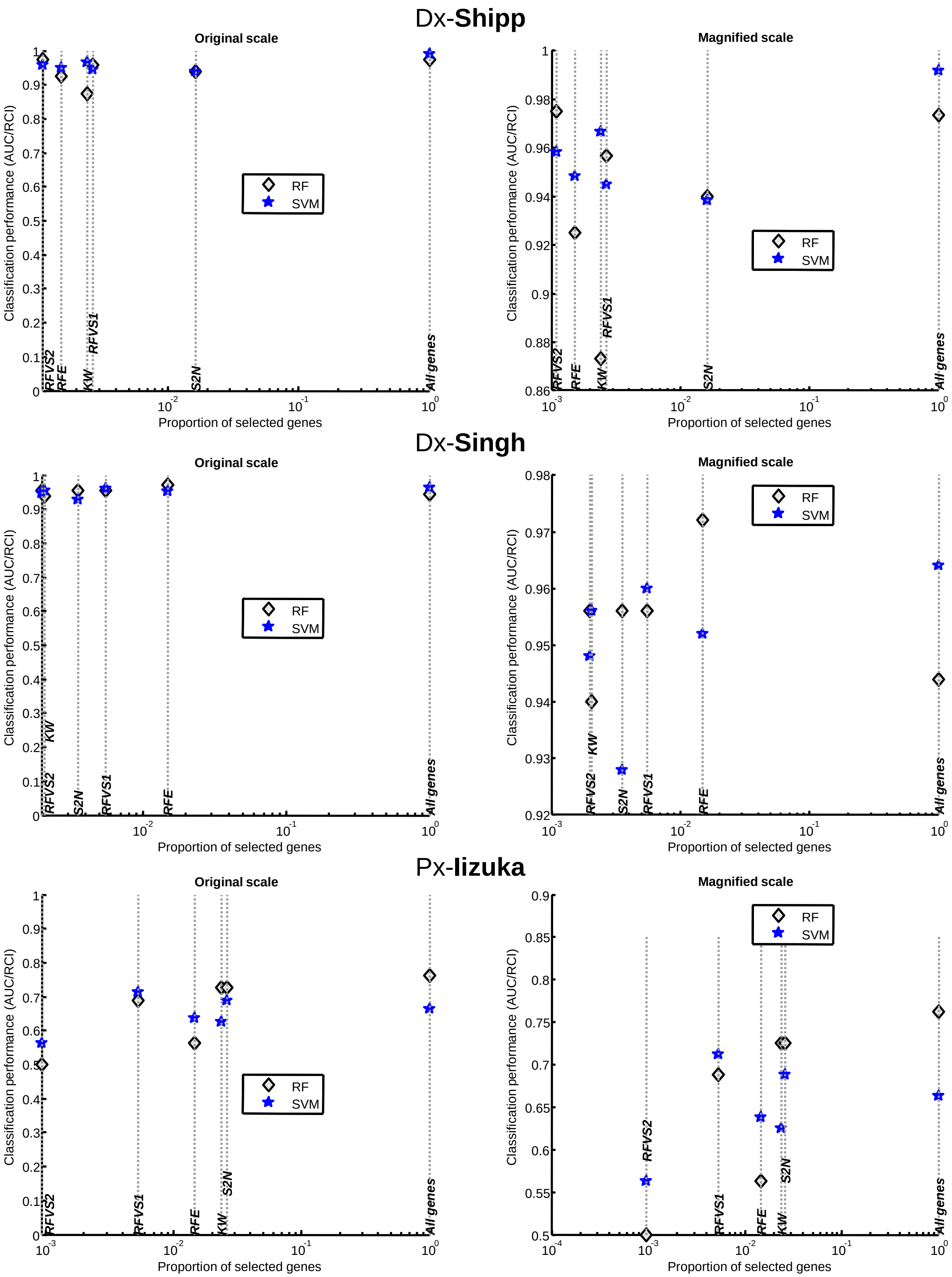
Dx-Shipp
Original scale
Magnified scale
Dx-Singh
Original scale
Magnified scale
Px-Iizuka
Original scale
Magnified scale
RF
SVM
Classification performance (AUC/RCI)
Proportion of selected genes
RFVS2
RFVS1
RFE
KW
S2N
All genes

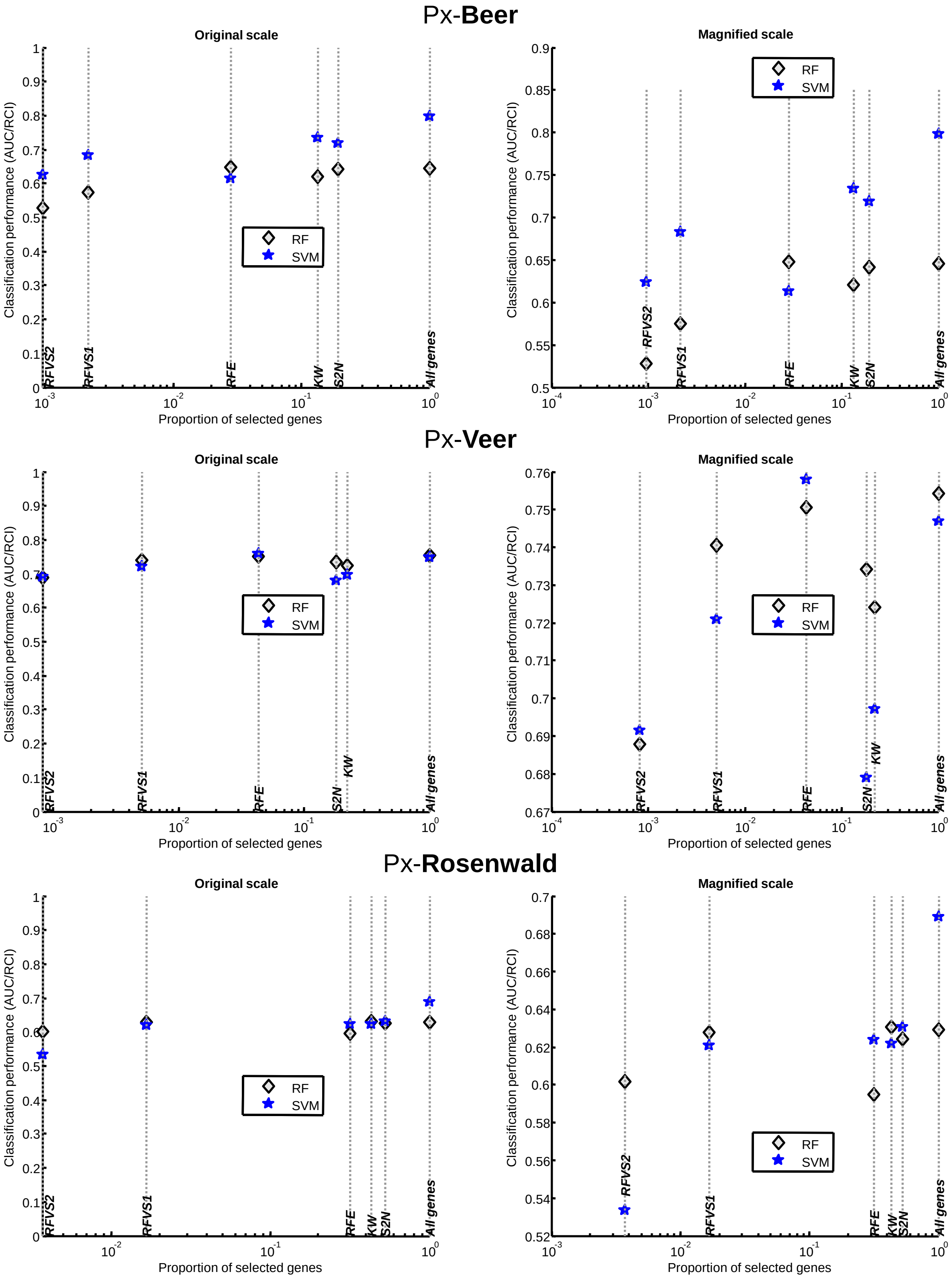

Px-Beer
Px-Veer
Px-Rosenwald
Original scale
Magnified scale
Classification performance (AUC/RCI)
Proportion of selected genes
RF
SVM
RFVS2
RFVS1
RFE
KW
S2N
All genes

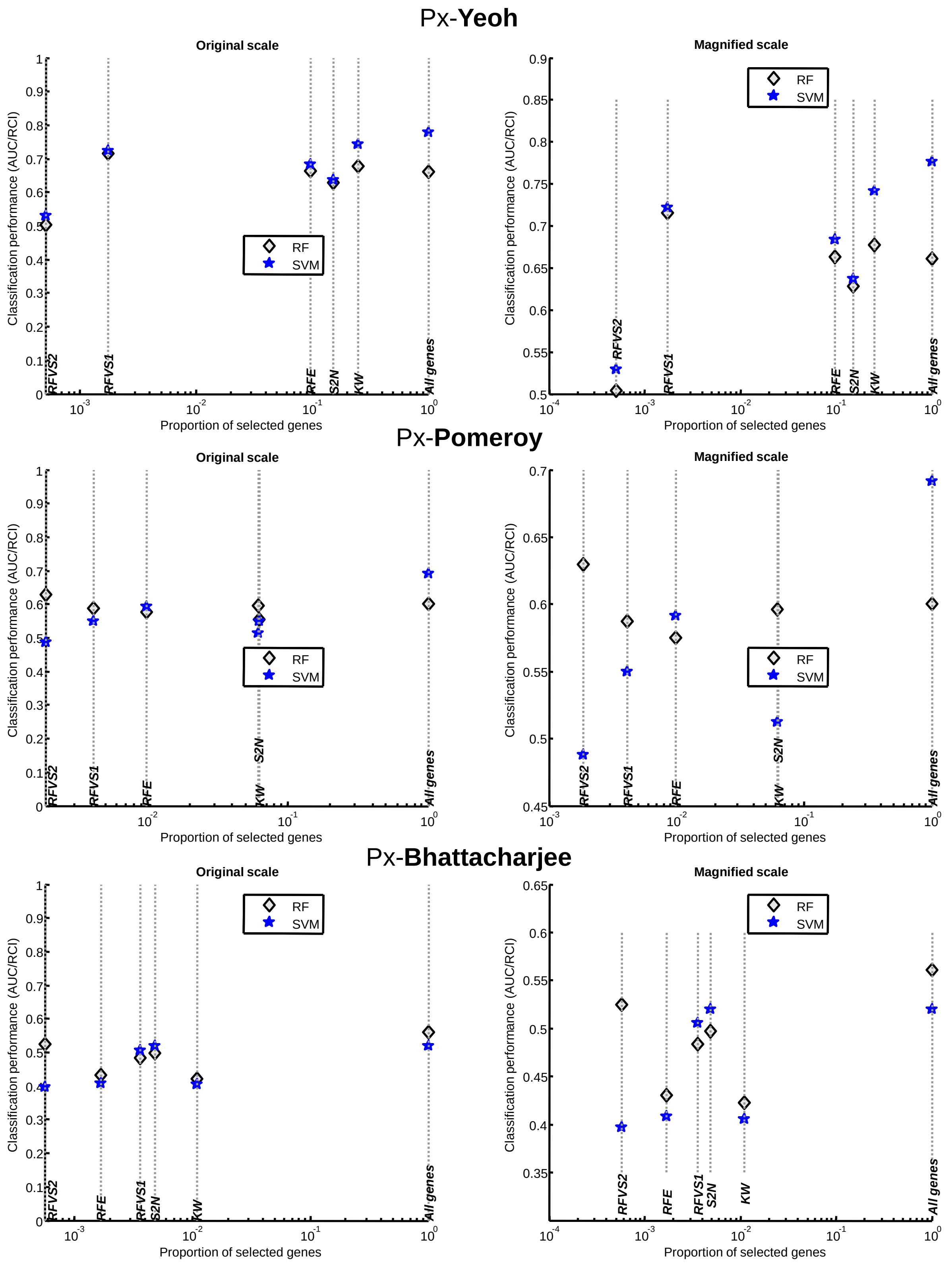
Px-Yeoh
Original scale
Magnified scale
Px-Pomeroy
Original scale
Magnified scale
Px-Bhattacharjee
Original scale
Magnified scale
RF
SVM
Classification performance (AUC/RCI)
Proportion of selected genes
RFVS2
RFVS1
RFE
S2N
KW
All genes

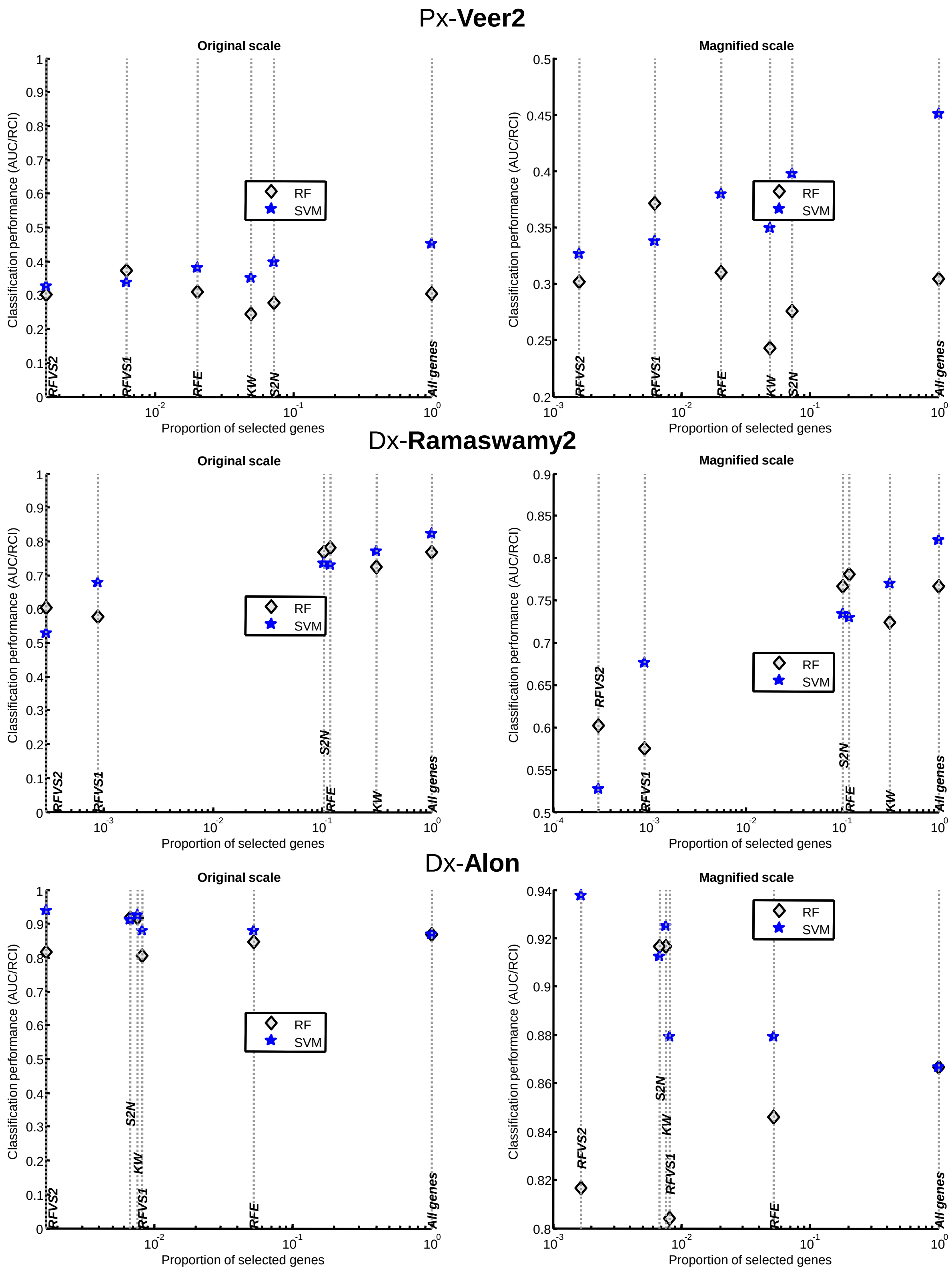
Px-Veer2
Original scale
Magnified scale
Dx-Ramaswamy2
Original scale
Magnified scale
Dx-Alon
Original scale
Magnified scale
Classification performance (AUC/RCI)
Proportion of selected genes
RF
SVM
RFVS2
RFVS1
RFE
KW
S2N
All genes

Dx-**Alizadeh**

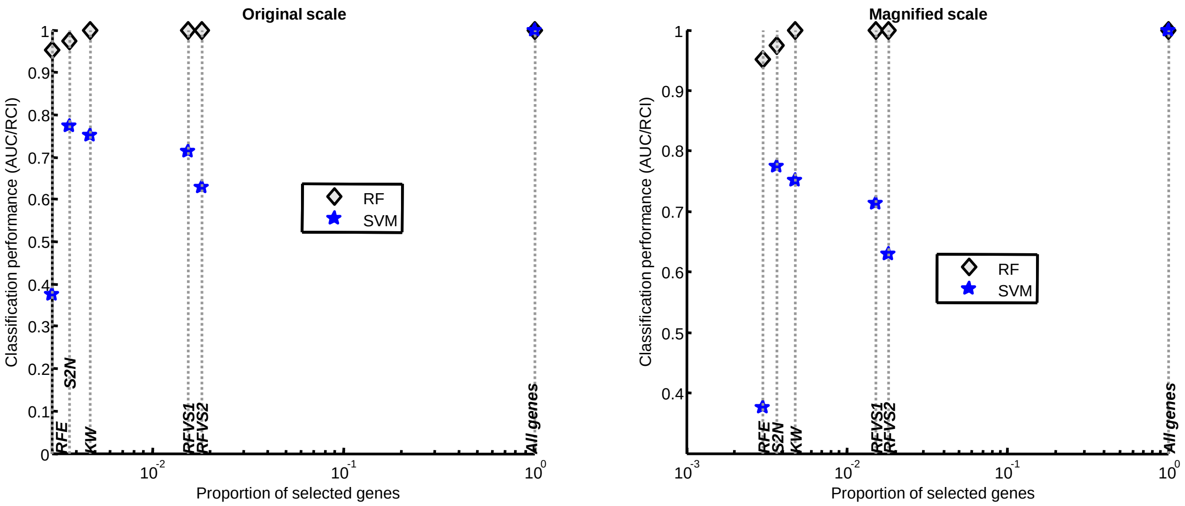

Original scale
Magnified scale
Classification performance (AUC/RCI)
Proportion of selected genes
RF
SVM
RFE
S2N
KW
RFVS1
RFVS2
All genes
