# Supplementary material for: A comprehensive comparison of random forests and support vector machines for microarray-based cancer classification
Source: BMC Bioinformatics. 2008 Jul 22;9:319. doi: 10.1186/1471-2105-9-319 (PMC2492881; doi:10.1186/1471-2105-9-319)
Supplement: Additional file 2 — Simulation experiment demonstrating sensitivity of random forests to input parameters. [file 1471-2105-9-319-S2.pdf]

Additional File 2:

Simulation experiment demonstrating sensitivity of random forests to input parameters.

Using a uniform random number generator with range [-1, 1], a dataset with 100 variables and 1,000 samples was generated. The response variable was constructed as the sign of sum of variables #1-#10 (i.e., it is a linear decision function). The dataset was randomly split into a non-overlapping training and testing set with 500 samples each.

Linear SVM classifier was applied to this dataset with penalty parameter *C* = {0.1, 1, 10 100, 1000, 10000}. RF classifier was applied with parameters *mtry* = {1, 2, 5, 10, 20} and *ntree* = {1, 10, 100, 1000, 10000}. The classification performance measured by AUC is provided in the tables below.

Performance of SVMs

C = 0.1	1	10	100	1000	10000
0.970	0.973	0.970	0.970	0.970	0.970

Performance of RFs

mtry	*ntree* = 1	10	100	1000	10000
1	0.502	0.628	0.722	0.861	0.880
2	0.572	0.678	0.815	0.905	0.913
5	0.548	0.725	0.879	0.936	0.947
10	0.552	0.789	0.909	0.957	0.956
20	0.631	0.775	0.924	0.949	0.951

As can be seen, for some parameters of RFs, the classification performance can be close to flipping a fair coin (0.5 AUC). On the other hand, SVMs are robust to the choice of penalty parameter *C*.
