# Supplementary material for: A comprehensive comparison of random forests and support vector machines for microarray-based cancer classification
Source: BMC Bioinformatics. 2008 Jul 22;9:319. doi: 10.1186/1471-2105-9-319 (PMC2492881; doi:10.1186/1471-2105-9-319)
Supplement: Additional file 3 — Complete information about microarray datasets used in the study. [file 1471-2105-9-319-S3.pdf]

Additional File 3:

Complete information about microarray datasets used in the study.

Task & dataset	*Number of classes*	*Number of genes*	*Number of samples*	*Prediction task*	*Reference*
Dx-Alizadeh	3	4026	62	Diffuse large B-cell lymphoma, follicular lymphoma, chronic lymphocytic leukemia	[1]
Dx-Alon	2	2000	62	Colon tumors and normal tissues	[2]
Dx-Armstrong	3	11225	72	AML, ALL and mixed-lineage leukemia (MLL)	[3]
Dx-Bhattacharjee	5	12600	203	4 lung cancer types and normal tissues	[4]
Dx-Golub	3	5327	72	Acute myelogenous leukemia (AML), acute lymphoblastic leukemia (ALL) B-cell and ALL T-cell	[5]
Dx-Khan	4	2308	83	Small, round blue cell tumors of childhood	[6]
Dx-Nutt	4	10367	50	4 malignant glioma types	[7]
Dx-Pomeroy	5	5920	90	5 human brain tumor types	[8]
Dx-Ramaswamy	26	15009	308	14 various human tumor types and 12 normal tissue types	[9]
Dx-Ramaswamy2	2	13247	76	Metastatic and primary tumors	[10]
Dx-Shipp	2	5469	77	Diffuse large B-cell lymphomas and follicular lymphomas	[11]
Dx-Singh	2	10509	102	Prostate tumor and normal tissues	[12]
Dx-Staunton	9	5726	60	9 various human tumor types	[13]
Dx-Su	11	12533	174	11 various human tumor types	[14]
Px-Beer	2	7129	86	Lung adenocarcinoma survival	[15]
Px-Bhattacharjee	2	12600	62	Lung adenocarcinoma 4-year survival	[4]
Px-Iizuka	2	7070	60	Hepatocellular carcinoma 1-year recurrence-free survival	[16]
Px-Pomeroy	2	7129	60	Medulloblastoma survival	[8]
Px-Rosenwald	2	7399	240	Non-Hodgkin lymphoma survival	[17]
Px-Veer	2	24188	97	Breast cancer 5-year metastasis-free survival	[18]
Px-Veer2	3	24188	115	Breast cancer 5-year metastasis-free survival, metastasis within 5 years, germline BRCA1 mutation	[18]
Px-Yeoh	2	12240	233	Acute lymphocytic leukemia relapse-free survival	[19]

References

1. Alizadeh AA, Eisen MB, Davis RE, Ma C, Lossos IS, Rosenwald A, Boldrick JC, Sabet H, Tran T, Yu X, Powell JI, Yang L, Marti GE, Moore T, Hudson J, Jr., Lu L, Lewis DB, Tibshirani R, Sherlock G, Chan WC, Greiner TC, Weisenburger DD, Armitage JO, Warnke R, Levy R, Wilson W, Grever MR, Byrd JC, Botstein D, Brown PO, Staudt LM: **Distinct types of diffuse large B-cell lymphoma identified by gene expression profiling**. *Nature* 2000, **403:**503-511.

2. Alon U, Barkai N, Notterman DA, Gish K, Ybarra S, Mack D, Levine AJ: **Broad patterns of gene expression revealed by clustering analysis of tumor and normal colon tissues probed by oligonucleotide arrays**. *Proc Natl Acad Sci U S A* 1999, **96:**6745-6750.

3. Armstrong SA, Staunton JE, Silverman LB, Pieters R, den Boer ML, Minden MD, Sallan SE, Lander ES, Golub TR, Korsmeyer SJ: **MLL translocations specify a distinct gene expression profile that distinguishes a unique leukemia**. *Nat Genet* 2002, **30:**41-47.

4. Bhattacharjee A, Richards WG, Staunton J, Li C, Monti S, Vasa P, Ladd C, Beheshti J, Bueno R, Gillette M, Loda M, Weber G, Mark EJ, Lander ES, Wong W, Johnson BE, Golub TR, Sugarbaker DJ, Meyerson M: **Classification of human lung carcinomas by mRNA expression profiling reveals distinct adenocarcinoma subclasses**. *Proc Natl Acad Sci U S A* 2001, **98:**13790-13795.

5. Golub TR, Slonim DK, Tamayo P, Huard C, Gaasenbeek M, Mesirov JP, Coller H, Loh ML, Downing JR, Caligiuri MA, Bloomfield CD, Lander ES: **Molecular classification of cancer: class discovery and class prediction by gene expression monitoring**. *Science* 1999, **286:**531-537.

6. Khan J, Wei JS, Ringner M, Saal LH, Ladanyi M, Westermann F, Berthold F, Schwab M, Antonescu CR, Peterson C, Meltzer PS: **Classification and diagnostic prediction of cancers using gene expression profiling and artificial neural networks**. *Nat Med* 2001, **7:**673-679.

7. Nutt CL, Mani DR, Betensky RA, Tamayo P, Cairncross JG, Ladd C, Pohl U, Hartmann C, McLaughlin ME, Batchelor TT, Black PM, von DA, Pomeroy SL, Golub TR, Louis DN: **Gene expression-based classification of malignant gliomas correlates better with survival than histological classification**. *Cancer Res* 2003, **63:**1602-1607.

8. Pomeroy SL, Tamayo P, Gaasenbeek M, Sturla LM, Angelo M, McLaughlin ME, Kim JYH, Goumnerova LC, Black PM, Lau C: **Prediction of central nervous system embryonal tumour outcome based on gene expression**. *Nature* 2002, **415:**436-442.

9. Ramaswamy S, Tamayo P, Rifkin R, Mukherjee S, Yeang CH, Angelo M, Ladd C, Reich M, Latulippe E, Mesirov JP, Poggio T, Gerald W, Loda M, Lander ES, Golub TR:

Multiclass cancer diagnosis using tumor gene expression signatures. *Proc Natl Acad Sci U S A* 2001, **98:**15149-15154.

10. Ramaswamy S, Ross KN, Lander ES, Golub TR: **A molecular signature of metastasis in primary solid tumors**. *Nat Genet* 2003, **33:**49-54.

11. Shipp MA, Ross KN, Tamayo P, Weng AP, Kutok JL, Aguiar RC, Gaasenbeek M, Angelo M, Reich M, Pinkus GS, Ray TS, Koval MA, Last KW, Norton A, Lister TA, Mesirov J, Neuberg DS, Lander ES, Aster JC, Golub TR: **Diffuse large B-cell lymphoma outcome prediction by gene-expression profiling and supervised machine learning**. *Nat Med* 2002, **8:**68-74.

12. Singh D, Febbo PG, Ross K, Jackson DG, Manola J, Ladd C, Tamayo P, Renshaw AA, D'Amico AV, Richie JP, Lander ES, Loda M, Kantoff PW, Golub TR, Sellers WR: **Gene expression correlates of clinical prostate cancer behavior**. *Cancer Cell* 2002, **1:**203-209.

13. Staunton JE, Slonim DK, Coller HA, Tamayo P, Angelo MJ, Park J, Scherf U, Lee JK, Reinhold WO, Weinstein JN, Mesirov JP, Lander ES, Golub TR: **Chemosensitivity prediction by transcriptional profiling**. *Proc Natl Acad Sci U S A* 2001, **98:**10787-10792.

14. Su AI, Welsh JB, Sapinoso LM, Kern SG, Dimitrov P, Lapp H, Schultz PG, Powell SM, Moskaluk CA, Frierson HF, Jr., Hampton GM: **Molecular classification of human carcinomas by use of gene expression signatures**. *Cancer Res* 2001, **61:**7388-7393.

15. Beer DG, Kardia SL, Huang CC, Giordano TJ, Levin AM, Misek DE, Lin L, Chen G, Gharib TG, Thomas DG, Lizyness ML, Kuick R, Hayasaka S, Taylor JM, Iannettoni MD, Orringer MB, Hanash S: **Gene-expression profiles predict survival of patients with lung adenocarcinoma**. *Nat Med* 2002, **8:**816-824.

16. Iizuka N, Oka M, Yamada-Okabe H, Nishida M, Maeda Y, Mori N, Takao T, Tamesa T, Tangoku A, Tabuchi H: **Oligonucleotide microarray for prediction of early intrahepatic recurrence of hepatocellular carcinoma after curative resection**. *Lancet* 2003, **361:**923-929.

17. Rosenwald A, Wright G, Chan WC, Connors JM, Campo E, Fisher RI, Gascoyne RD, Muller-Hermelink HK, Smeland EB, Giltnane JM, Hurt EM, Zhao H, Averett L, Yang L, Wilson WH, Jaffe ES, Simon R, Klausner RD, Powell J, Duffey PL, Longo DL, Greiner TC, Weisenburger DD, Sanger WG, Dave BJ, Lynch JC, Vose J, Armitage JO, Montserrat E, Lopez-Guillermo A, Grogan TM, Miller TP, LeBlanc M, Ott G, Kvaloy S, Delabie J, Holte H, Krajci P, Stokke T, Staudt LM: **The use of molecular profiling to predict survival after chemotherapy for diffuse large-B-cell lymphoma**. *N Engl J Med* 2002, **346:**1937-1947.

18. van't Veer LJ, Dai H, van de Vijver MJ, He YD, Hart AA, Mao M, Peterse HL, van der KK, Marton MJ, Witteveen AT, Schreiber GJ, Kerkhoven RM, Roberts C, Linsley

PS, Bernards R, Friend SH: **Gene expression profiling predicts clinical outcome of breast cancer**. *Nature* 2002, **415:**530-536.

19. Yeoh EJ, Ross ME, Shurtleff SA, Williams WK, Patel D, Mahfouz R, Behm FG, Raimondi SC, Relling MV, Patel A, Cheng C, Campana D, Wilkins D, Zhou X, Li J, Liu H, Pui CH, Evans WE, Naeve C, Wong L, Downing JR: **Classification, subtype discovery, and prediction of outcome in pediatric acute lymphoblastic leukemia by gene expression profiling**. *Cancer Cell* 2002, **1:**133-143.
